# Climbing behavior and growth response of *Mansoa alliacea* to various support forms in vertical greening

**DOI:** 10.3389/fpls.2025.1561073

**Published:** 2025-04-08

**Authors:** Piao Jiang, Jinxin Zhu, Shufei Weng, Zhuoran Li, Feican Xu

**Affiliations:** ^1^ School of Forestry and Landscape Architecture, South China Agricultural University, Guangzhou, China; ^2^ School of Architecture, South China University of Technology, Guangzhou, China

**Keywords:** lianas, *Mansoa alliacea*, support poles, climbing nets, climbing growth, biomass allocation

## Abstract

**Introduction:**

With rapidly expanding urban environments, green space is becoming increasingly limited. Vertical greening serves as a viable solution to this challenge, with climbing plants playing a crucial role in creating functional and aesthetic green structures. However, current research on support structures for lianas remains scarce. This study therefore investigates the impact of various support forms on the growth of *Mansoa alliacea*, a potential species for vertical greening.

**Methods:**

One-year-old Mansoa alliacea seedlings were exposed to eight types of support systems: bamboo poles and PVC pipes with diameters of 8 mm and 24 mm; and climbing nets made of nylon and hemp with mesh sizes of 5×5 cm and 10×10 cm. This study evaluated the effects of these supports on plant morphology, photosynthetic parameters, and biomass allocation.

**Results and discussion:**

The results showed that climbing nets promoted better above-ground growth, measured as number of leaves, leaf area, stem length, and internode length, while poles more effectively enhanced root branching. Various supports forms influenced biomass distribution. Climbing nets tended to allocate more biomass to aboveground parts, while support poles tended to allocate more biomass to underground parts. Bamboo poles (8 mm diameter) and hemp nets (10×10 cm mesh size) were found to be the most effective. These results suggest prioritizing rough climbing nets like hemp nets as structural supports for *Mansoa alliacea* to promote rapid vertical green landscape formation.

## 1 Introduction

Urbanization has driven significant economic growth and social development, but it has also created serious environmental challenges, such as air pollution, urban heat islands, and a loss of biodiversity (Zaid et al., 2018; Wu et al., 2023). Simultaneously, the expansion of urban areas has drastically reduced available green space, increasing the need for innovative solutions to enhance urban ecological sustainability (Rupasinghe and Halwatura, 2020). As awareness of these environmental issues grows, vertical greening has emerged as a promising strategy to mitigate the negative impacts of urbanization. By utilizing building facades to support vegetation, vertical greening not only contributes to environmental benefits such as air purification and energy savings but also improves urban aesthetics and the well-being of residents (Dominici et al., 2022; Wang et al., 2022).

Climbing plants are essential components of vertical greening systems due to their ability to thrive in limited space. They attach to vertical surfaces using specialized climbing mechanisms, which expands greening opportunities in densely built environments (Koyama et al., 2013; Wang et al., 2022). However, their successful integration into urban landscapes depends on the availability of suitable support systems. The type, material, and configuration of support systems are critical factors influencing plant growth, biomass allocation, and overall performance. While considerable research has been conducted on the climbing mechanisms and growth strategies of climbing plants (Isnard and Silk, 2009; Gianoli, 2015; Rodriguez-Quintero et al., 2022), the specific effects of support system characteristics (e.g., diameter, material, and mesh size) on biomass allocation and photosynthetic efficiency in *Mansoa alliacea* remain poorly understood.

Support structures significantly influence biomass allocation between the shoot and root systems of climbing plants, with varying effects on plant growth depending on the type of support used. Research indicates that support structures can affect biomass allocation patterns in climbing plants by facilitating vertical growth and canopy expansion (Feitosa et al., 2023). For instance, twining plants like *Wisteria floribunda* show increased stem elongation and total biomass but reduced root biomass when provided with external support (Sakai and Suzuki, 1999). However, the response of climbing plants to different support structures is species-specific. For example, *Wisteria floribunda* allocates more biomass to stems when supported by poles, while *Celastrus orbiculatus* does not show any significant change when grown on trellises (Wyka et al., 2019a). This highlights the importance of using support structures that reflect the specific needs of each climbing plant species.

Climbing plants select support structures based on factors like material, shape, and stability. Studies have shown that the diameter, height, and structure of support trees, as well as their growth rate and bark type, can influence plant preference for different support systems (Roeder et al., 2015; Mori et al., 2016). Climbing plants use different attachment strategies, such as twining or tendrils, to reduce stem construction costs and gain a competitive advantage in light capture (Darwin, 1867; Isnard and Silk, 2009). However, biomechanical constraints limit the suitability of different supports depending on the plant’s climbing mechanism. Tendril climbers, in particular, can use a narrower range of support diameters compared to twining climbers. If the support size exceeds their limit, they cannot attach securely and may slip off (Carrasco-Urra and Gianoli, 2009; Durigon et al., 2013).

Although the effects of support structures on climbing plants have been studied to some extent, comparing the growth responses of climbing species—especially woody ones—to different support systems is limited. Some studies have investigated factors like support diameter (Tao et al., 2003; Zhu et al., 2018), angle (Tao and Zhong, 2003), and the effects of climbing nets (Jiao, 2016), and found significant differences in growth patterns, plasticity, and biomass allocation between plants with and without supports. Selecting appropriate support structures is thus critical for optimizing climbing plant growth (Gianoli, 2015). However, there is still little known about the effects of different support types on the growth of *Mansoa alliacea*, a potential species for vertical greening.

This study aims to address this gap by examining the effects of several different support structures on the growth of *Mansoa alliacea*, a promising liana used in vertical greening systems. Eight different support systems were tested, including bamboo poles and PVC pipes with diameters of 8 mm and 24 mm, and climbing nets made from nylon and hemp with mesh sizes of 5×5 cm and 10×10 cm. The study focused on the morphological, photosynthetic, and biomass characteristics of plants grown with these support structures and analyzed how each support influenced plant adaptation. The study findings can provide valuable insight for selecting optimal support systems that promote the growth of lianas in vertical greening applications, contributing to the development of more effective and sustainable urban greening strategies

## 2 Materials and methods

### 2.1 Materials and methods

#### 2.1.1 Plant materials and growth conditions

*Mansoa alliacea* was selected as the focus of this study, as it is a tendril-based liana with strong potential for landscape greening. The experiment was conducted at the nursery of South China Agricultural University in Guangzhou, Guangdong Province, China (23°9′28″N, 113°21′7″E). It is situated within the South Asian subtropical monsoon climate zone, characterized by ample sunlight and abundant rainfall, an annual mean temperature ranging from 21.4 to 21.9°C, average annual precipitation between 1623.6 and 1899.8 mm, and 1820–1960 annual sunshine hours.

**Figure 1:**
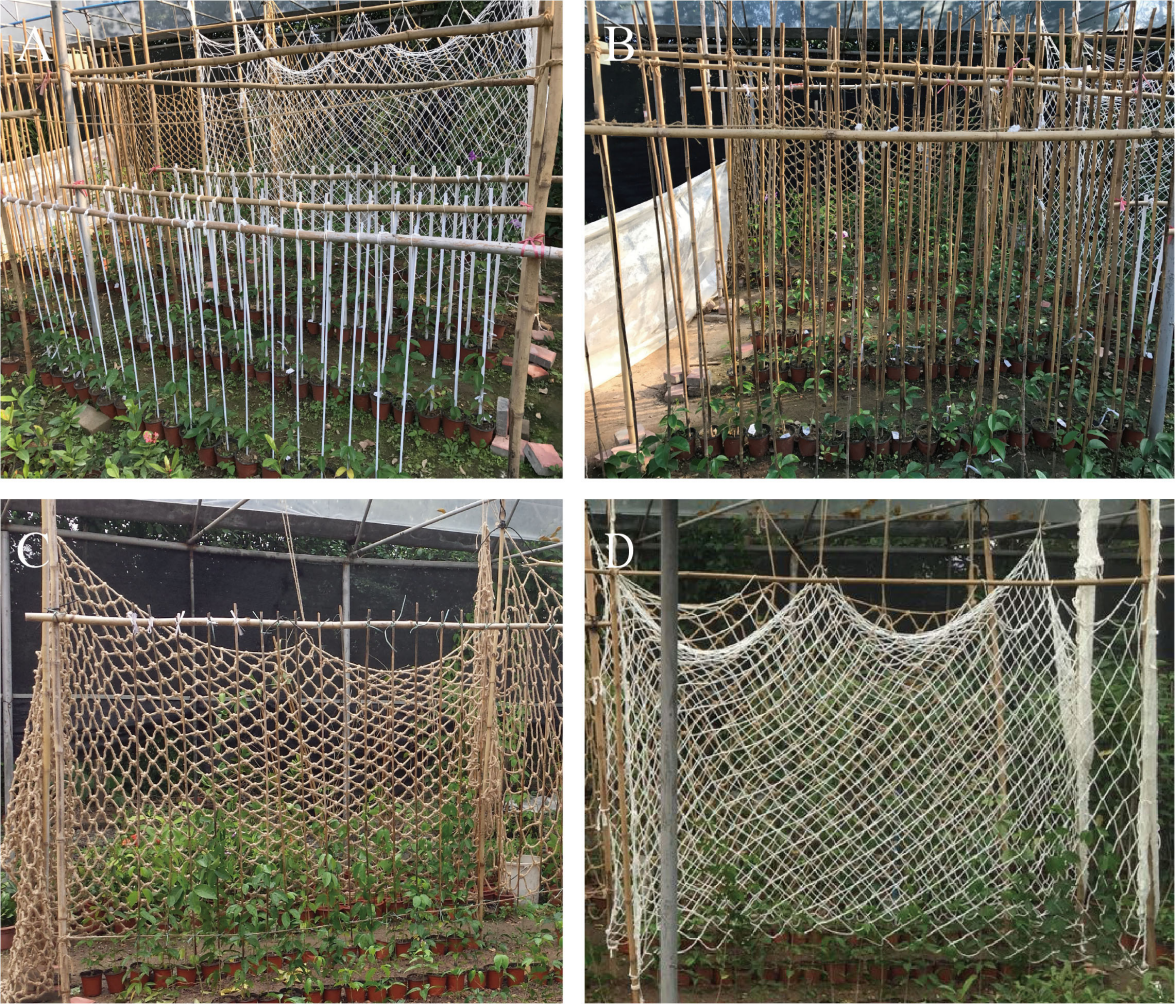
Field setup of four types of supports. **(A)** Setup of PVC pipe support poles with different diameters; **(B)** Setup of bamboo rod support poles with different diameters; **(C)** Setup of climbing nets made of hemp rope with different mesh sizes; **(D)** Setup of climbing nets made of nylon rope with different mesh sizes.

On October 22, 2016, uniform one-year-old *Mansoa alliacea* cuttings were transplanted into pots (12 cm deep and 15 cm in diameter) filled with a soil mixture. The experimental treatments included 8 types of supports: bamboo poles and PVC pipes of 8 mm and 24 mm diameters each, and hemp climbing nets and nylon climbing nets with mesh sizes of 5×5 cm and 10×10 cm each (**Figure 1**). These treatments were labeled as B8, B24, P8, P24, H5, H10, N5, and N10, respectively. Each climbing net was 2 meters high and 1 meter wide, with strands of 6 mm thickness fixed 20 cm above each pot’s base. The selection of these materials and specifications was based on the research by Zhu et al. (2018) and Jiao (2016) on different specifications of support poles and climbing nets, as well as the common use of bamboo poles and PVC pipes as support structures for vine seedlings in nurseries in southern China. In urban landscaping, nylon and hemp nets with varying mesh sizes are frequently used for constructing plant trellises. Based on these practical applications, the materials selected for this experiment were chosen for their availability and suitability for promoting use in landscaping. The experiment included eight treatments, and each was repeated five times with four plants per replicate.

During the initial growth phase, plants were manually guided to attach to the supports. Plants that could not naturally attach were tied to the supports then allowed to grow freely. All plants were maintained under identical irrigation and fertilization regimes. Data collection for morphological, biomass, and physiological measurements began on January 5, 2017.

### 2.2 Experimental measurements

#### 2.2.1 Morphological parameters

Morphological traits were recorded for each plant throughout the experiment. Leaf number (LN), shoot number (SN), tendril number (TN), stem length (SL, cm), and internode length (IL, cm) were measured with a measuring tape or ruler (accuracy: 1 mm). Total leaf area (TLA, cm²) was estimated using WinFolia 2008 software. Branches were defined as extensions longer than 10 cm with at least two leaves, and fully unfolded leaves were counted as valid. Root systems were washed and separated from stem tissues, then scanned, and analyzed using the WanShen LA-S root analysis system to quantify root length (RL, cm), root surface area (RSA, cm²), and root branch number (RSN).

#### 2.2.2 Biomass measurements

Roots, stems, leaves, and tendrils were oven-dried at 80°C for 48 hours. Their respective dry weights -root biomass (RB, g), stem biomass (SB, g), leaf biomass (LB, g), and tendril biomass (TB, g)- were measured using an electronic balance (accuracy: 0.0001 g). Aboveground biomass (AGB, g) was calculated as the sum of stem, leaf, and tendril biomasses. Total biomass (TB, g) was calculated as the sum of AGB and RB, while the root-to-shoot ratio (R/S) was calculated as RB/AGB.

#### 2.2.3 Photosynthetic physiological parameters

Photosynthetic characteristics were measured using an LI-6400 portable photosynthesis system (LI-COR, USA). Mature, healthy leaves from the upper parts of plants were selected for measuring net photosynthetic rate (Pn, μmol·m^-2^·s^-1^), stomatal conductance (Gs, mmol·m^-2^·s^-1^), intercellular CO_2_ concentration (Ci, μmol·mol^-1^), and transpiration rate (Tr, mmol·m^-2^·s^-1^). Measurements were taken between 9:00 AM and 11:00 AM, as this is a period of active photosynthesis. Ten readings were averaged per leaf under conditions of 1000 μmol·mm^-2^·s^-1^ light intensity, room temperature, 500 μmol·s^-1^ airflow, and ambient CO_2_ concentration. Chlorophyll content was determined using acetone extraction, following Gao Junfeng (Gao, 2006).

#### 2.2.4 Statistical analysis

Mann-Whitney U tests were employed for pairwise comparisons. For multi-group comparisons, one-way analysis of variance (ANOVA) and Duncan’s multiple range test were used to identify significant differences among treatments. Pearson correlation analysis was applied to evaluate relationships between aboveground and root traits. Statistical analyses were conducted using IBM SPSS Statistics 26, and figures were created using Origin 2022. Data were reported as mean ± standard error (SE).

## 3 Results

### 3.1 Effect of different support structures on the morphological growth of *Mansoa alliacea*

**Figure 2:**
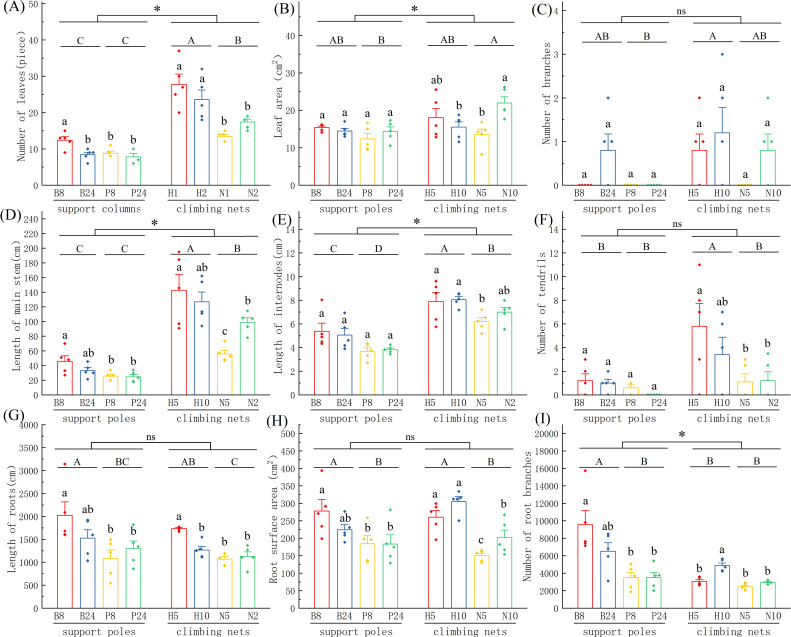
Comparison of the morphological traits of *Mansoa alliacea* with different support types. **(A)** Leaf number, **(B)** Leaf area, **(C)** Branch number, **(D)** Stem length, **(E)** Internode length, **(F)** Tendril number, **(G)** Root length, **(H)** Root surface area, **(I)** Number of root branches. Different uppercase and lowercase letters denote significant differences at *P* < 0.05; *indicates significant differences at *P* < 0.05 between the support pole group and the climbing net group; ns indicates no significant difference between the two groups.

The comparative analysis of support structures on *Mansoa alliacea* morphology shows that the specifications, materials, and types of support structures significantly affect plant growth (as shown in **Figure 2**). In the comparison of support pole specifications, plants supported by B8 performed significantly better than those supported by P8 and P24 across several indicators, including leaf number, main stem length, root length, root surface area, and the number of root branches. However, no significant difference was observed between B8 and B24 plants. In the comparison of climbing net specifications, no significant differences were found in the aboveground morphology between plants supported by H5 and H10. However, the underground morphology showed a clear advantage for H5 plants in terms of root length and the number of root branches, which were significantly higher than those of H10 plants (*P* < 0.05). In the nylon rope net group, N10 plants exhibited significantly higher leaf area, main stem length, and root surface area than N5 plants (*P* < 0.05), suggesting that larger mesh sizes in nylon rope nets are more favorable for the growth of *Mansoa alliacea*.

In the comparison of support materials, plants supported by hemp rope nets (H5 and H10) showed significant increases in leaf number, main stem length, internode length, tendril number, root length, and root surface area compared to those supported by nylon rope nets (N5 and N10) (*P* < 0.05). This suggests that hemp rope nets provide more ideal climbing conditions for *Mansoa alliacea* compared to nylon rope nets. In the comparison of support pole materials, bamboo poles (B8 and B24) significantly outperformed PVC pipes (P8 and P24) in terms of internode length, root length, root surface area, and the number of root branches (*P* < 0.05), suggesting that relatively rough materials are more favorable for the growth of climbing plants.

In the overall comparison of support types, climbing nets promoted significant aboveground growth of *Mansoa alliacea*, particularly in terms of leaf number, leaf area, main stem length, and internode length. The performance of plants supported by climbing nets was superior to that of pole-supported plants to a significant degree (*P* < 0.05). In contrast, support poles were more advantageous for measures of root growth, particularly the number of root branches, which was significantly higher in the support pole group than in the climbing net group (*P* < 0.05). However, no significant differences were found between the two support types in terms of root length and root surface area. These results suggest that climbing nets are more effective than support poles in enhancing the aboveground growth of *Mansoa alliacea*. Plants supported by climbing nets howed better climbing performance, longer main stems, longer internodes, and more tendrils, all of which contribute to faster vegetative spread and reproduction.

### 3.2 Effect of different support structures on photosynthetic physiology

The results of the analysis examining different support structures’ effects on photosynthetic physiological parameters of *Mansoa alliacea* (as shown in **Table 1**) indicate significant differences between the support pole group and the climbing net group. In the support pole group, plants supported by B8 exhibited significantly higher net photosynthetic rate and chlorophyll content (*P* < 0.05), showing stronger light energy absorption compared to other treatment groups. In contrast, plants supported by PVC pipes had significantly lower chlorophyll content than those supported by bamboo poles. Furthermore, the stomatal conductance of B24 plants was significantly lower than that of P24 plants, while no significant difference was observed between stomatal conductance of B8 and P8 plants, suggesting a similar effect on this particular measurement.

**Table 1:** Multiple comparisons of photosynthetic physiological indices of *Mansoa alliacea* under different support structure types.

Treatment Groups	Pn/(μmol·m^-2^·s^-1^)	Gs/(mmol·m^-2^·s^-1^)	Ci/(mol·m^-2^·s^-1^)	Tr/(μmol·mol^-1^)	Chl/(mg·g^-1^)
B8	4.55 ± 0.05a	0.08 ± 0.01ab	307.76 ± 14.01ab	2.92 ± 0.00b	2.76 ± 0.05a
B24	4.32 ± 0.04b	0.06 ± 0.01b	274.17 ± 20.83bc	2.92 ± 0.00b	2.42 ± 0.01b
P8	4.14 ± 0.04c	0.10 ± 0.01a	339.67 ± 13.43a	2.92 ± 0.00b	2.08 ± 0.13c
P24	4.15 ± 0.04c	0.10 ± 0.02a	339.42 ± 12.41a	2.93 ± 0.00b	1.99 ± 0.05cd
H5	4.52 ± 0.01a	0.05 ± 0.00b	250.00 ± 6.94c	2.93 ± 0.00b	1.67 ± 0.09d
H10	4.50 ± 0.05a	0.05 ± 0.00b	268.99 ± 9.92bc	2.93 ± 0.00b	2.13 ± 0.08bc
N5	4.40 ± 0.05ab	0.07 ± 0.01ab	300.10 ± 9.30abc	2.95 ± 0.00a	1.97 ± 0.06cd
N10	4.29 ± 0.11bc	0.06 ± 0.01b	261.60 ± 31.09bc	2.94 ± 0.00a	1.83 ± 0.05cd

B8 - 8 mm bamboo rods; B24 - 24 mm bamboo rods; P8 - 8 mm PVC pipes; P24 - 24 mm PVC pipes; H5 - 5×5 cm hemp rope nets; H10 - 10×10 cm hemp rope nets; N5 - 5×5 cm nylon rope nets; N10 - 10×10 cm nylon rope nets. Pn, net photosynthetic rate; Gs, stomatal conductance; Ci, intercellular CO_2_ concentration; Tr, transpiration rate; Chl, Chlorophyll content. Different lowercase letters in the same pole indicate significant differences between treatments (*P*<0.05).

In the climbing net group, plants supported by N10 had significantly lower net photosynthetic rates compared to other treatment groups (*P* < 0.05), indicating that larger mesh sizes in nylon rope nets may limit the photosynthetic efficiency of *Mansoa alliacea*. In contrast, plants supported by hemp rope nets exhibited significantly higher net photosynthetic rates compared to those supported by nylon rope nets (*P* < 0.05), while their transpiration rates were significantly lower than those in the nylon rope net group (*P* < 0.05). This suggests that hemp rope nets provide a more favorable growing environment and promote more efficient photosynthesis in *Mansoa alliacea*. Additionally, no significant differences were observed between the two net groups in terms of stomatal conductance and intercellular CO_2_ concentration.

Plants supported by H10 exhibited significantly higher chlorophyll content than those supported by H5 (*P* < 0.05), indicating that larger mesh sizes in hemp rope nets help increase chlorophyll content in *Mansoa alliacea*. However, no significant differences in chlorophyll content were found between N5 and N10 treatment groups.

### 3.3 Effects of different support structures on biomass accumulation and allocation

**Figure 3:**
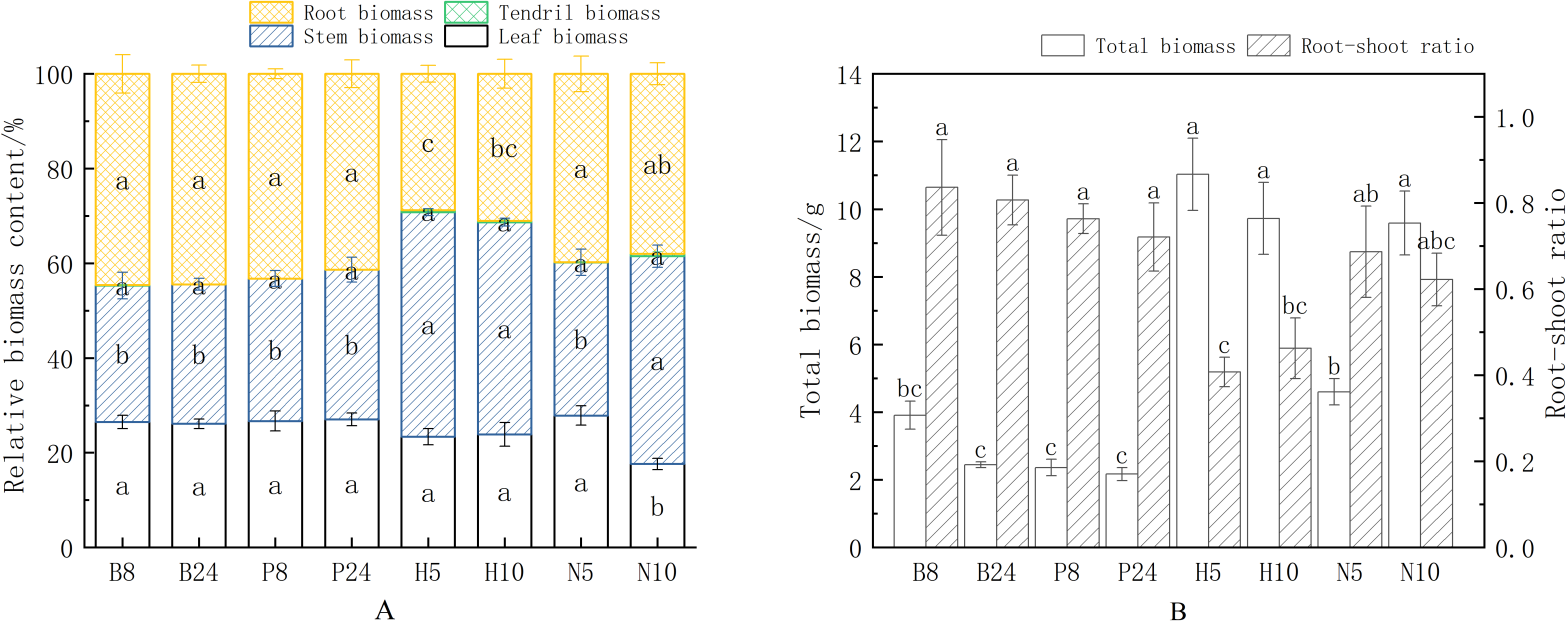
Biomass allocation and accumulation of *Mansoa alliacea* under different support types. **(A)** Biomass allocation percentages of leaves, stems, roots, and tendrils in *Mansoa alliacea* biomass. Different lowercase letters indicate significant differences in the biomass ratios of the same organ at *P* < 0.05. **(B)** Total biomass and root-to-shoot ratio of *Mansoa alliacea*. Different lowercase letters in the same row indicate significant differences between treatments at *P* < 0.05.

The results of the analysis indicate that support structure changes had a significant influence on the patterns of biomass distribution in *Mansoa alliacea* (as shown in **Figure 3A**), but the diameter and material of the support poles did not influence the ratio of biomass allocation between roots, stems, and leaves. Specifically, plants supported by poles tended to allocate more biomass to their roots, whereas plants supported by climbing nets prioritized biomass allocation to their stems. Among them, the stem biomass ratios of H5, H10, and N10 were significantly higher than those of the plants on support poles (*P* < 0.05), while the leaf biomass ratio of N10 was significantly lower than that of other treatment groups (*P* < 0.05). The leaf, stem, and root biomass ratios of N5 showed no significant difference compared to the support pole group. Furthermore, Since the seedlings were still in the early growth stage and tendril numbers were low, there was no significant difference in tendril biomass ratios between the treatments. This difference in allocation between aboveground and underground parts may be an outcome of the variable effects of different support structures on plant growth requirements. The material of the climbing nets was also significantly associated with differences in plant biomass accumulation and distribution pattern. The root biomass ratio of H5 and H10 was significantly lower than that of N5 (*P* < 0.05), while the stem biomass ratio was significantly higher than that of N5 (*P* < 0.05). This suggests that the hemp rope net helps promote stem elongation in *Mansoa alliacea*, increasing the proportion of biomass allocated to the stem.

From the root-to-shoot ratio (as shown in **Figure 3B**), the root-to-shoot ratios of H5 and H10 were significantly lower than those of the support pole treatment group (*P* < 0.05), indicating a significant regulatory effect on the distribution of resources between the aboveground and underground parts for plants using hemp rope nets compared to support poles. Although there was no significant difference in the root-to-shoot ratios between N5, N10, and the support pole group, the ratios were still slightly lower in the climbing net groups. This suggests that *Mansoa alliacea*, when supported with a climbing net, invests more biomass into aboveground parts, whereas when climbing with a support pole, it invests more biomass into underground parts to enhance root anchorage. In terms of total biomass accumulation, H5, H10, and N10 had significantly higher total biomass accumulation than the support pole group. Although the total biomass of N5 showed no significant difference compared to the support pole group, it was still slightly higher. This indicates that, compared to the support pole group, the climbing nets are more effective in promoting the total biomass accumulation of *Mansoa alliacea*.

### 3.4 Correlation analysis between aboveground and underground traits

**Figure 4:**
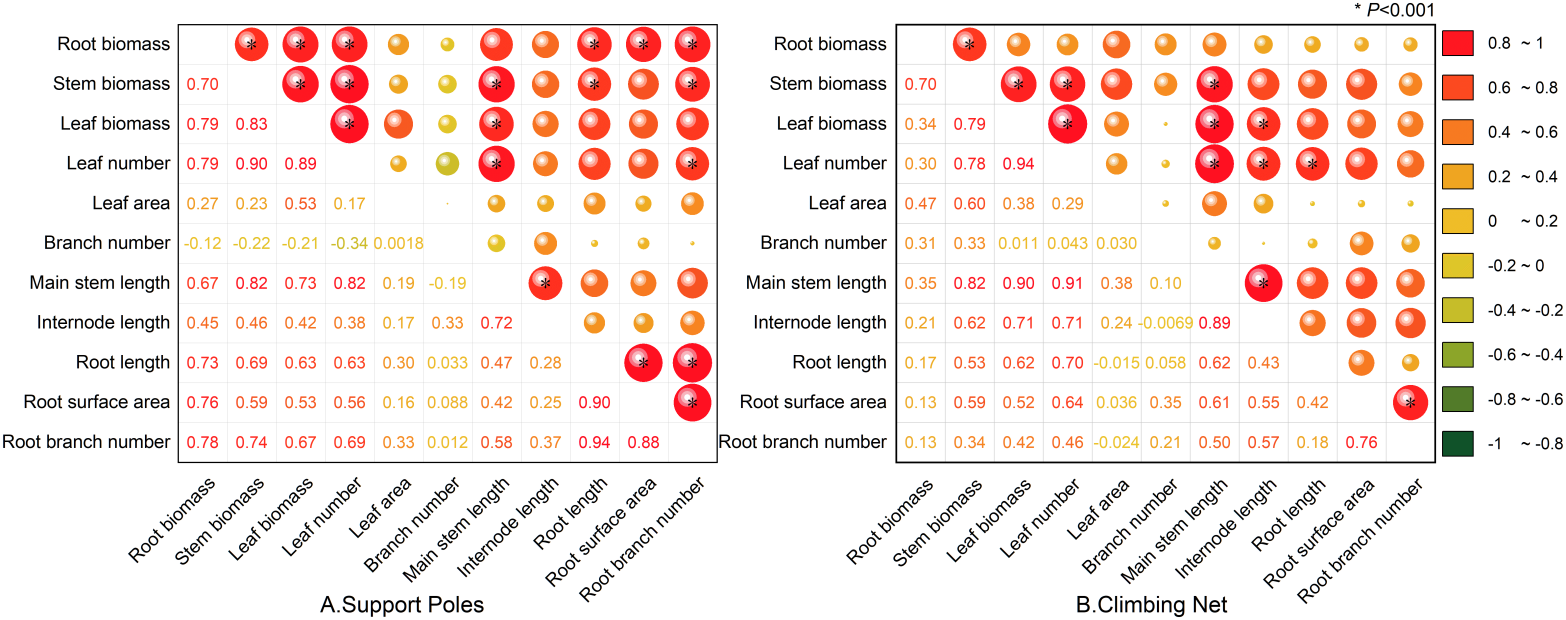
Correlation between morphology and biomass of *Mansoa alliacea* under climbing net and support pole conditions. **(A)** Correlation between morphology and biomass under the support pole condition. **(B)** Correlation between morphology and biomass under the climbing net condition. *, indicates significant correlation at *P* < 0.001.

Based on the correlation analysis of the aboveground and underground morphological and biomass characteristics of *Mansoa alliacea* in the support pole group (as shown in **Figure 4A**), there was a significant positive correlation between root length, surface area, and branching number (*P* < 0.001). This indicates a close relationship between root extension, root surface area expansion, and root branching density. In the correlation analysis between underground and aboveground morphology, the number of root branches showed a significant positive correlation with both stem length and leaf number (*P* < 0.001), suggesting that the branching density of the roots is closely related to the growth of aboveground parts. In terms of biomass correlation, the leaf, stem, and root biomasses all showed significant, positive correlations with one another (*P* < 0.001), indicating that biomass accumulation in the aboveground and underground parts is interrelated.

In the correlation analysis of the climbing net group (as shown in **Figure 4B**), the number of root branches was significantly and positively correlated with root surface area (*P* < 0.001), while root length showed a significant and positive correlation with leaf number (*P* < 0.001), suggesting that root extension may have a certain relationship with leaf growth. Additionally, root biomass was significantly positively correlated with stem biomass and that stem biomass was significantly positively correlated with leaf biomass (*P* < 0.001). This shows that the biomass accumulation in roots, stems, and leaves is tightly interconnected.

Further analysis of the correlation between aboveground morphology in the climbing net and support pole groups (as shown in **Figures 4A, B**) revealed that stem length was significantly positively correlated with leaf number and internode length (*P* < 0.001), whereas branching number had no significant correlation with the morphology and biomass of either the aboveground or underground parts. This may be related to the fact that *Mansoa alliacea* has fewer branches during its early growth stage. It has not yet entered a rapid branching phase. Additionally, it may be related to the plant’s strong apical dominance, which limits natural branching.

## 4 Discussion

This study investigates the effects of different support structures on the climbing growth of *Mansoa alliacea* and compares the results with previous research. The findings indicate that the diameter, material, and surface properties of support structures have a significant influence on the climbing growth, biomass allocation, and photosynthetic capacity of climbing plants. The study thus offers valuable theoretical insight that may be used in the design of optimal support structures for vertical greening systems.

### 4.1 The effect of support diameter on climbing growth

The results of this study show that an 8 mm bamboo pole was more effective than a 24 mm bamboo pole in supporting the climbing growth of *Mansoa alliacea*, particularly in terms of root development and stem elongation. Consistent with Rjosk et al.’s (2018), our findings highlight the critical role of support diameter in climbing performance, suggesting that smaller diameters enhance tendril attachment and climbing efficiency. Similarly, Carrasco-Urra and Gianoli (2009) and Durigon et al. (2013) found that twining and tendril-bearing climbing plants have specific support diameter limits, beyond which attachment becomes unstable and climbing ability is hampered. In this study, the plants supported by the 8 mm bamboo pole exhibited superior growth compared to those supported by the 24 mm pole, further demonstrating the critical role of support diameter in climbing performance. In landscaping practice, small-diameter support poles are similar to ropes and steel wires, making them suitable for guiding the growth of twining and tendril-type climbing plants.

In the comparison of climbing nets, this study found no significant differences in the aboveground morphology of plants supported by 5×5 cm and 10×10 cm hemp rope nets. However, plants supported by the 10×10 cm hemp rope net outperformed those supported by nylon nets, suggesting that material properties of the support structure may have a more substantial impact than the mesh size. In summary, *Mansoa alliacea* can effectively climb on the 10x10cm hemp rope and nylon rope climbing nets. This is consistent with Jiao’s (2016) experimental results on the climbing network of different specifications of *Pyrostegia venusta* and *Passiflora edulis*, showing that the 10×10 cm climbing net could promotes the growth of plant height, leaf number, tendril length, and branching, and further improves photosynthesis and biomass accumulation of the vines.

### 4.2 The impact of support material on plant growth

The material properties of support structures significantly influence the climbing growth of vine plants. Previous studies have shown that the surface characteristics of support materials, such as the roughness of tree bark, can affect the pole selection of climbing plants. Rough bark structures facilitate climbing, while smooth or flaky bark may cause the plant to slip (Roeder et al., 2015). Furthermore, the surface friction of the support material plays a crucial role in the attachment strength of climbing plants. Previous studies have found that ivy can only effectively climb textured or porous surfaces like wood, cork, or mortar, and fails to attach to smooth surfaces like glass or aluminum (Melzer et al., 2012). The roughness of the support surface seems to increase the friction between the tendrils of climbing plants and the support structure, thereby enhancing attachment strength and promoting climbing growth.

In this study, bamboo poles were more effective than smooth PVC pipes in supporting the climbing growth of *Mansoa alliacea*, particularly in terms of root development and biomass allocation. This finding is consistent with studies by Fiorello et al. (2020) and Rjosk et al. (2018), who noted that rougher surfaces like bamboo significantly increase the friction between tendrils and the support structure compared to smooth surfaces like aluminum poles, thereby enhancing climbing performance. Of the climbing net materials examined in this study, the 5×5 cm and 10×10 cm hemp rope nets outperformed nylon rope nets in promoting plant growth. This difference may be related to the fact that the hemp rope net has a rougher surface than the nylon rope.

In urban landscaping, Bamboo poles and PVC pipes are commonly used for constructing garden trellises and supporting climbing plants. Steel wire mesh and metal frames are commonly used for high-rise buildings and plants with heavier loads, while hemp rope nets and nylon rope nets are suitable for light support structures, such as building facades and landscape green walls, offering advantages in cost and installation. Although this study did not conduct a detailed comparison of the climbing net materials, it is speculated that climbing nets with higher surface roughness can promote plant attachment and growth. Therefore, in landscaping practice, natural and rough-surfaced hemp rope nets can be prioritized. Future research should further explore the impact of different materials on plant growth to select the most suitable support structures for optimal aesthetic and functional outcomes.

### 4.3 The effect of support structure on photosynthesis

This study also explored the impacts of support structure on the photosynthesis of *Mansoa alliacea*. Chlorophyll content is an important indicator of photosynthetic capacity, with higher net photosynthetic rates typically associated with stronger stomatal control and enhanced accumulation of photosynthetic products (Lambers et al., 2008; Croft et al., 2017). Our study found that the net photosynthetic rate and chlorophyll content of *Mansoa alliacea* increased significantly under more effective climbing conditions, suggesting that suitable support structures can enhance the plant’s ability to absorb and convert light energy. Specifically, plants supported by smaller diameter poles (e.g., 8 mm bamboo poles) and rough-textured climbing nets (e.g., hemp rope nets) exhibited significantly higher photosynthetic activity, indicating that selecting the appropriate support structure can improve light resource utilization. Moreover, the H10 group had significantly higher chlorophyll content compared to the H5 group, supporting Jiao’s (2016) findings that plants supported by 10×10 cm climbing nets had superior photosynthetic capacity. However, plants in the N5 group, which showed poor climbing performance, did not exhibit significantly different photosynthetic rates compared to those in the H5 and H10 groups, suggesting that the relationship between climbing performance and photosynthetic parameters is not always straightforward.

### 4.4 Biomass allocation in relation to support structure

Biomass allocation, which entails the distribution of resources among the plant’s leaves, stems, and roots, is a key metric for determining overall plant growth and adaptability. The results of this study revealed no significant differences in biomass allocation between the different support treatments, but did find significant differences in biomass accumulation in both the supporting structures and assimilatory organs (*P* < 0.05). This may be due to the early growth stage of the *Mansoa alliacea* individuals used in this study, with resource allocation differences yet to be pronounced. In the different support groups, more biomass was allocated to the roots which led to a higher root-to-shoot ratio. This may have reduced the plant’s competitive ability for aboveground resources. Wyka et al. (2019b) suggested that climbing plants typically allocate more biomass to aboveground parts when they are supported, and this enhances climbing ability. In this study, plants supported by climbing nets allocated more biomass to their aboveground parts, particularly to the stems, which helped promote stem elongation and branching and thus improved climbing ability.

### 4.5 Aboveground and underground biomass allocation

The relationship between aboveground and underground biomass allocation plays a critical role in resource acquisition and adaptation to environmental changes (Poorter et al., 2011). The results of this study indicate that suitable climbing net conditions significantly enhance root branching, expanding the root surface area, increasing soil contact, and improving water and nutrient uptake. This in turn drives biomass accumulation in the plant’s aboveground parts (e.g., leaves and stems). The length of the main stem is determined by internode length and number, with aboveground biomass growth primarily driven by stem elongation and branching. This process facilitates climbing and improves light capture, enhancing the overall effectiveness of vertical greening. Additionally, this study found a significant correlation between leaf number and root length. According to the optimal allocation theory (OPT), when underground resources like water and nutrients are limited, plants allocate more biomass to their roots to enhance resource acquisition (Wang et al., 2023). However, this trade-off generally results in reduced biomass allocation to stems and leaves, which is also necessary for the plant’s basic survival (Wang et al., 2023).

Seedling growth is staged. Although this study provides valuable insights into the effects of different support structures on the growth and biomass allocation of *Mansoa alliacea*, the results are limited by factors such as weather and experimental conditions. The study only reflects the patterns of early-stage climbing growth, as the duration of the experiment was relatively short. This limitation affects our comprehensive understanding of the long-term growth patterns and biomass allocation dynamics of climbing plants and prevents a full evaluation of how biomass changes with plant size. The allometric growth method can reveal how biomass allocation changes with plant size and the potential trade-offs in biomass distribution between organs at different developmental stages (Poorter et al., 2011). Feitosa et al. (2023) also demonstrated that the allometric growth model can be used to assess the differences in size, allocation, and investment of climbing plants based on support structures. Therefore, future research integrating allometric modeling could thus provide deeper ecological and physiological insights into climbing plant adaptation strategies in vertical greening systems.

## 5 Conclusions

This study demonstrated that support structures have a significant impact on the climbing growth of lianas. The study confirms prior research on the effects of different support structure forms, materials, and size on liana morphology, biomass accumulation and distribution, and photosynthetic physiology. Specifically, climbing nets were more effective than support poles in promoting the growth of the aboveground parts of *Mansoa alliacea*, including leaf number, leaf area, main stem length, internode length, branching, and tendril number, while also significantly increasing the number of root branches. The material and mesh size of the climbing net had a significant impact on biomass distribution, with plants supported by climbing nets allocating more biomass to the aboveground parts, especially as stem growth, thereby improving their climbing ability. In contrast, variations in the diameter and material of support poles did not result in significant differences in biomass allocation between roots, stems, and leaves but did promote more biomass investment in the root system to enhance anchorage ability. Among the support pole treatments, bamboo poles were more effective than PVC pipes, and the 8mm diameter bamboo pole provided the most optimal support for *Mansoa alliacea.* In terms of climbing net material, hemp rope nets outperformed nylon rope nets, with the 10x10cm mesh size being the most effective. Based on these findings, it is recommended that landscaping applications prioritize the use of natural, relatively rough hemp rope nets as support structures for *Mansoa alliacea* to facilitate the rapid development of vertical greening.

## Data Availability

The original contributions presented in the study are included in the article material. Further inquiries can be directed to the corresponding author.
